# Flow Injection Photosensitized Chemiluminescence of Luminol with Cu(II)-Rose Bengal: Mechanistic Approach and Vitamin A and C Determination

**DOI:** 10.1155/2014/109592

**Published:** 2014-12-28

**Authors:** Muhammad Asgher, Mohammad Yaqoob, Abdul Nabi, Ghulam Murtaza, Abdul Rauf Siddiqi, Amir Waseem

**Affiliations:** ^1^Department of Chemistry, University of Balochistan, Sariab Road, Quetta 87300, Pakistan; ^2^Department of Pharmacy, COMSATS Institute of Information Technology, Abbottabad 22060, Pakistan; ^3^Department of Biosciences, COMSATS Institute of Information Technology, Islamabad 45320, Pakistan; ^4^Department of Chemistry, Quaid-i-Azam University, Islamabad 45320, Pakistan

## Abstract

Rose Bengal photosensitized flow injection chemiluminescence method is reported using luminol-Cu(II) for the determination of vitamins A and C in pharmaceutical formulations. The reaction is based on the enhancement effect of analyte in the production of anion radicals of Rose Bengal (RB^**•**−^) which rapidly interact with dissolved oxygen and generate superoxide anions radicals (O_2_^**•**−^) and hydrogen peroxide (H_2_O_2_). Highly reactive hydroxyl radicals (^**•**^OH) were produced via dismutation of H_2_O_2_ by catalyst (Cu^2+^). The generated superoxide anions radicals and hydroxyl radicals thus oxidize luminol in alkaline medium to generate strong chemiluminescence. The limit of detection (3*s* of the blank, *n* = 6) of vitamins A and C and RB was found to be 0.008, 0.005, and 0.05 *μ*g mL^−1^, respectively. The sample throughput of 70 h^−1^ for vitamins A and C and 30 h^−1^ for RB was found. Calibration curve was linear in the range of 0.05–15, 0.01–20, and 0.1–50 *μ*g mL^−1^ for vitamins A and C and RB, respectively, with relative standard deviations (RSDs; *n* = 3) in the range 1.6–3.6%. The method was successfully applied to pharmaceutical formulations and the results obtained were in good agreement with the labeled values.

## 1. Introduction

Vitamins are organic substances present in several foods in low quantities and are indispensable to organism functions. Their systematic absence in the diet can result in deficient growing and development. Vitamin A is a necessary micronutrient in the diet for vision, growth, reproduction, and maintenance of the immune system. Humans require only minute amount of vitamin A in their diets (400 to 1300 *μ*g of retinol equivalents per day, depending on age and sex) [1]. Vitamin A deficiency can result in blindness and is associated with increased risk of severe infection and death. Vitamin C is the generic descriptor for all compounds exhibiting qualitatively the biological activity of ascorbic acid. Ascorbic acid is important for several physiological functions and involves redox characteristics that allow ascorbic acid to play an important role in the antioxidant protection of cells. Thus, ascorbic acid represents the major water-soluble antioxidant in plasma and tissues [1]. Rose Bengal (RB) or Acid Red 94 (4,5,6,7-tetrachloro-2′,4′,5′,7′-tetraiodofluorescein) is a xanthine dye and its sodium salt is commonly used in eye drops to stain damaged conjunctival and corneal cells and thereby identify damage to the eye. RB has been used to assess hepatic function and ophthalmic disorders and has been used as a photosensitiser in treatment of skin lesions and photothermal and photodynamic oral cancer therapies [2–4]. Luminol is one of the most commonly used CL reagents for the determination of various analytes in diverse matrices. A number of oxidants have been used for luminol reactions, for example, permanganate, hypochlorite, and iodine, and the most useful oxidant is hydrogen peroxide. Transition metal ions (Co^2+^, Cu^2+^, Fe^3+^, etc.) are used as a typical catalyst for luminol CL reactions [5]. Different analytical techniques have been reported in literature for the determination of vitamin A (most often retinol) in diverse samples including flow injection spectrophotometry [6], chemiluminescence [7–10], and HPLC [11–13]. Few flow injection chemiluminescence methods are reported for the determination of vitamin C in pharmaceuticals, urine, and blood serum [14–16]. RB is being used for number of applications including various analyte determinations [17–20] and Firefly luciferin-activated RB chemiluminescence is also used for the photodynamic therapy of cancer [2]. The spectrophotometric method for RB determination in water samples using liquid-liquid extraction is reported recently [21].

## 2. Experimental

### 2.1. Materials and Reagents

All glassware and bottles were cleaned overnight in 2% v/v nutrient free detergent (Neutracon, Decon Laboratories Ltd., Hove, UK), rinsed with ultrahigh purity (UHP) water (0.0167 *μ*S cm^−1^, Elga, Purelab Option, UK), followed by soaking in 10% v/v hydrochloric acid overnight, again rinsed with UHP water, and stored in plastic bags to prevent contamination. All chemicals used were of analytical reagent grade and solutions were prepared in UHP water. Retinol, retinyl acetate, retinyl palmitate, *α*-tocopherol, *α*-tocopheryl acetate, and vitamin D stock solutions (100 mg L^−1^) were prepared by dissolving 5.0 mg of each compound in 50 mL methanol using dark brown volumetric flasks. These solutions were stable for at least one month when stored in the dark at −20°C. Working solutions were prepared from these stock solutions by appropriate dilution with sodium pyrophosphate buffer (pH 10.25, 0.01 M) and protected from light.

Luminol stock solution (0.01 M) was prepared by dissolving 0.178 g of luminol (5-amino-2,3-dihydrophthalazine-1,4-dione) in 25 mL of 0.01 M sodium hydroxide, sonicated for 10 min, made up of 100 mL with UHP water, and stored in a polyethylene volumetric flask at 4°C. The working luminol solution (5 × 10^−5^ M) was prepared by diluting 1.0 mL of the stock solution to 200 mL with 0.125 mol L^−1^ sodium hydroxide. This solution was stored for at least 24 h to allow removal of trace metal impurities from the alkaline reagent via adsorption on to the walls of the reagent bottle and used as required.

Copper(II) stock solution (0.01 M) was prepared by dissolving the required amount of copper nitrate in 0.1 M HCl. RB stock solution (1 × 10^−3^ M) was prepared daily by dissolving the required amount in 100 mL water and stored at room temperature. A mixture solution of RB (1 × 10^−4^ M) and copper nitrate (5 × 10^−4^ M) was prepared daily by diluting required volume of these stock solutions with pyrophosphate buffer (pH 10.25, 0.01 M).

For the interference study, stock solutions (500 mg L^−1^) of *β*-carotene, vitamins K_1_, K_2_, and D_3_, cholesterol, oleic acid, and stearic acid were prepared in methanol, stored in the refrigerator at 4°C, and protected from the light. Sucrose, glucose, starch, lactose, and glycerol solutions were prepared in UHP water. Working standard solutions were prepared from these stock solutions in sodium pyrophosphate buffer (pH 10.25, 0.01 M) as required for the interference study.

### 2.2. Apparatus and Procedures

**Figure 1:**
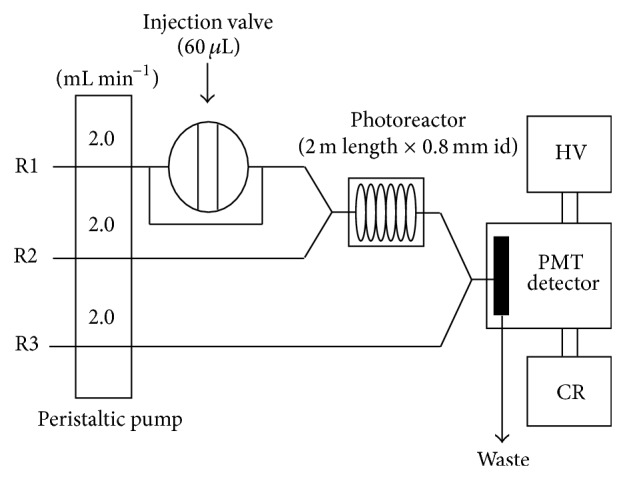
FI-CL manifold for the determination of retinol. R1 = pyrophosphate buffer (0.01 M, pH 10.25), R2 = RB (1 × 10^−4^ M) containing Cu^2+^ (5 × 10^−4^ M), R3 = luminol solution (5 × 10^−5^ M), CR = chart recorder, HV = high voltage power supply, and PMT = photomultiplier tube detector.

The FI-CL manifold used in this work is shown in Figure 1. A peristaltic pump (four channels, Ismatec Reglo, Switzerland) was used to propel all solutions at a flow rate of 2.0 mL min^−1^ (each channel). A six-way rotary injection valve (Rheodyne 5020, Anachem, Luton, UK) equipped with a 60 *μ*L sample loop was used to introduce samples into pyrophosphate buffer (pH 10.25, 0.01 M) stream (R1 in Figure 1). Sample carrier stream was merged with RB (1 × 10^−4^ M) containing copper nitrate (5 × 10^−4^ M), prepared in pyrophosphate buffer (pH 10.25, 0.01 M) (R2 in Figure 1). The merged streams were then passed through a 200 cm photoreactor and then merged at a T-piece with luminol (5 × 10^−5^ M in NaOH 0.125 M) stream (R3 in Figure 1) and allowed to pass through a glass spiral flow cell (1.5 mm i.d., 18 mm dia.), positioned in front of an end window photomultiplier tube (9798B, Electron Tubes Ltd., Ruislip, UK). The PMT, glass coil, and T-piece were enclosed in a light-tight housing. The high voltage for PMT was set to 1000 V via a 2 kV power supply (MP20SN, Thorn EMI, UK). The CL signal generated was recorded using a chart recorder (BD 40, Kipp & Zonen, Holland). For RB determinations, reverse FIA is used where RB (R2 channel) is acting as an analyte solution and fixed amount (20 *μ*g/mL) of ascorbic acid is injected to get the calibration graph for RB.

### 2.3. Construction of Photoreactor

The photoreactor used in the proposed studies consisted of PTFE tubing (2.0 m length × 0.8 mm i.d., Fisher, UK) wound around a thin glass plate with exposed area (4.5 × 5.5 cm) placed on a compact 4 W UV lamp (Model UVGL-25, UVP, Upland, USA). The plate was placed at a distance of 1.0 cm from the lamp which produced ultraviolet radiation of wavelength 254 nm. The lamp, glass plate, and PTFE tubing were covered with aluminum foil. The process of photoreaction was carried out at room temperature.

### 2.4. Sample Preparation

The commercial pharmaceutical formulations containing vitamin A were analyzed by the proposed method. Initially, these samples were saponified by taking an amount of each sample and dissolved in 3 mL methanol in light protected glass tube covered with aluminum foil and then treated with KOH (3 mL, 60%). The mixture was incubated at 45–50°C for 2 h with intermittent mixing. The incubated sample was then extracted four times with n-hexane (5 mL). The organic layer was recovered and dried under nitrogen stream and the residues were redissolved in methanol. After appropriate dilution with pyrophosphate buffer (pH 10.25, 0.01 M), the samples were injected directly to the proposed FI-CL manifold for the determination of retinol.

## 3. Results and Discussion

### 3.1. Optimization of Reaction Conditions

To establish the optimum conditions for the determination of retinol, the effects of key chemical and physical parameters were investigated using a univariate approach. These include selection of a catalyst, pyrophosphate buffer pH, reagents concentrations (buffer, RB, copper(II), sodium hydroxide, and luminol), flow rates, sample volume, and photoreactor length that were optimized for maximum CL intensity and the results are summarized in Table 1. All these studies were performed with a 2.5 *μ*g mL^−1^ retinol standard solution (60 *μ*L) and a PMT voltage of 1000 V.

#### 3.1.1. Selection of Metal as a Catalyst

It has been reported that RB has the ability to generate oxidizing species via metal ion catalyzed free radical decomposition of hydrogen peroxide [22]. Since the radical anion of RB is a reducing species, it may react with redox active metal ions such as copper or iron under aerobic conditions and form superoxide anion and hydrogen peroxide. In the proposed CL system, copper(II) was acting as a catalyst and the possible reduction to copper(I) in the presence of retinol and RB possibly enhances the CL signal. Therefore, the effect of various metal ions (Fe(II and III), V(III and IV), Cr(III and VI), Mn(II and VII), Ni(II), Cu(II), Co(II), and Pb(II)) was studied for their catalytic activities (5 × 10^−5^ M) on the proposed FI-CL system to obtain the highest CL signals with lower background. The most reproducible signals with the lowest background were observed when Cu(II) was used as a catalyst. Therefore, Cu(II) concentration was inevitable to be optimized. The effect of Cu(II) concentration was examined over the range of 1 × 10^−4^-1 × 10^−3^ M. The maximum CL response was observed at Cu(II) concentration of 5 × 10^−4^ M and therefore was selected and used for further studies.

#### 3.1.2. Effect of Pyrophosphate Buffer pH and Concentration

The efficiency of photoproducts by RB in the presence of analyte under UV light was highly dependent upon the pH of pyrophosphate buffer. The optimum pyrophosphate buffer pH was examined over the range of 8.5–11.0. An increase pyrophosphate buffer pH up to 10.25 resulted in a maximum CL response and further increase in pH gave decrease in CL response. Therefore, pyrophosphate buffer of pH 10.25 was selected and used for subsequent experiments. The pyrophosphate buffer concentration (pH 10.25) was then examined over the range of 0.001–0.05 M. Maximum CL intensity was observed at 0.01 M with reproducible signals and further increase in buffer concentration resulted in noisy CL signals and high background. Therefore, pyrophosphate buffer of pH 10.25 with concentration of 0.01 M was selected and used subsequently.

#### 3.1.3. Effect of RB Concentration

RB is a well-known and widely studied water-soluble photosensitizing dye of xanthine origin with a strong visible absorption band around 500–550 nm [23]. In the proposed CL system, RB is used as a photosensitizer and the effect of its concentration on CL intensity was examined over the range 1 × 10^−5^–2.5 × 10^−4^ M containing Cu(II) (5 × 10^−4^ M). An optimum CL intensity was observed at 1 × 10^−4^ M and further increase in RB concentration resulted in a decrease in CL response possibly due to self-absorption. The selected concentration of RB (1 × 10^−4^ M, manifold R2 channel) is further diluted by the solutions of incoming two other channels (R1 and R3) and the color intensity of RB further decreased to a point where it does not cause interference in CL signal. Pure RB dye (10^−6^ M to 10^−5^ M) has an absorption peak above 500 nm, whereas CL emission of luminol centered around 425 nm which is not affected by dilute RB solution.

#### 3.1.4. Effect of Sodium Hydroxide Concentration

It is well-known that the luminol CL reaction requires alkaline medium conditions. In the proposed CL system, various alkaline buffer solutions were examined for luminol, including carbonate, borate, and sodium hydroxide to improve the sensitivity of the system. Sodium hydroxide was found the most suitable medium for present CL system. Therefore, the effect of sodium hydroxide concentration was examined over the range 0.05–0.2 M. An optimum CL intensity was observed at 0.125 M sodium hydroxide, above which a decrease in CL intensity was observed with high background and noisy CL signals. Therefore, sodium hydroxide concentration of 0.125 M was used in all subsequent experiments.

#### 3.1.5. Effect of Luminol Concentration

The effect of luminol concentration was then examined over the range 1 × 10^−5^-1 × 10^−4^ M under the optimized sodium hydroxide solution (0.125 M). As the concentration was increased, both the CL intensity and background increased. The maximum CL intensity was observed at 5 × 10^−5^ M luminol. Therefore, luminol concentration of 5 × 10^−5^ M was used in the following experiments.

#### 3.1.6. Effect of Physical Parameters

The effect of key physical parameters including flow rates, sample volume, and photoreactor coil length was then investigated (Table 1). A flow rate of 2 mL min^−1^ (individual channel) gave the optimum CL intensity with reproducible peak height and was used for all the three reagent streams. A sample injection volume of 60 *μ*L and a photoreactor of 2 m length × 0.8 mm i.d. also gave optimum CL intensity and were used for all subsequent experiments. The optimum PMT voltage was 1000 V.

### 3.2. Possible CL Mechanism

Rose Bengal is a xanthine dye with outstanding photosensitizing ability [23], soluble in polar media, including water, strongly absorbs light (molar absorption coefficient of the maximum absorbance is as high as 10^5^ dm^3^ mol^−1^ cm^−1^), and, on photoexcitation, readily forms its triplet excited state. The yield for intersystem crossing for RB has been estimated very close to unity [24]:

(1) $$\begin{array}{c} \text{RB}+h\nu ⟶{\text{RB}}^{*}\left(\text{T}\right) \end{array}$$

The high efficiency of RB as a photosensitizer is because of the high yield of formation of the lowest triplet excited state of RB (RB^*^(T)) and its long intrinsic life time. It has been reported that RB can efficiently sensitize the generation of singlet oxygen (O_2_ (^1^Δ_g_)), a key oxidant in photosensitized oxidation reaction [25]:

(2) RB∗T+O2⟶O2(Δ1g)+RB

Although the generation of singlet oxygen in photosensitized reaction by RB is considered the predominant route of oxidation, it has also been reported that part of the energy of the triplet excited state of this dye is utilized in an electron transfer from RB^*^(T) to O_2_; as a result, a cation radical RB^•+^ and superoxide anion (O_2_^•−^) are formed:

(3) $$\begin{array}{c} {\text{RB}}^{*}\left(\text{T}\right)+{\text{O}}_{2}⟶{{\text{O}}_{2}}^{•-}+{\text{RB}}^{•+} \end{array}$$

If an excited molecule can accept electrons, another mode of photosensitized reaction should be considered, namely, the formation of anion radicals species (RB^•−^). These species can initiate a sequence of redox reactions involving many molecules which may not be susceptible to photoreactions. Electron transfer is the basis for such photosensitized oxidation reactions [26]. Xanthine dyes are known for their ability to undergo redox reaction; in the presence of appropriate electron donors, RB can be reduced easily to its bleached dihydro form [27]. It has been postulated that this two-electron reaction occurs in steps as two consecutive one-electron reactions, with the formation of the anion radical (RB^•−^) as an intermediate [28]:

(4) $$\begin{array}{c} \text{RB}+{\text{e}}^{-}\;\left(\text{electron}\;\text{donor}\right)⟶{\text{RB}}^{•-} \end{array}$$

The electron spin resonance (ESR) study has shown that anion radicals of Rose Bengal (RB^•−^, reducing radicals) formed during electron transfer reaction interact rapidly with molecular oxygen in an aerobic environment to form superoxide anion and, via its dismutation, hydrogen peroxide was formed. The hydrogen peroxide production has been shown to be increased with the increase in concentration of electron donor:

(5) $$\begin{array}{c} \text{RB}+{\text{e}}^{-}\;\left(\text{electron}\;\text{donor}\right)⟶{\text{RB}}^{•-}\\ {\text{RB}}^{•-}+{\text{O}}_{2}⟶{{\text{O}}_{2}}^{•-}+\text{RB}\\ {{2\text{O}}_{2}}^{•-}+2{\text{H}}^{+}⟶{\text{H}}_{2}{\text{O}}_{2}+{\text{O}}_{2} \end{array}$$

The ESR studies have also shown the reduction of copper by H_2_O_2_ and production of hydroxyl radicals [22]. The heterogenous catalyst (Cu) reduction by hydrogen peroxide in copper-mediated fenton reaction, its mechanism and production of hydroxyl radicals (OH^•^), dioxygen (O_2_), and water (H_2_O) have been thoroughly studied [29] and the mechanism has been formulated as

(6) $$\begin{array}{c} {\text{Cu}}^{2+}+{\text{H}}_{2}{\text{O}}_{2}⟶{\text{Cu}}^{+•}{\text{O}}_{2}\text{H}+{\text{H}}^{+}\\ {\text{Cu}}^{+•}{\text{O}}_{2}\text{H}+{\text{H}}_{2}{\text{O}}_{2}⟶{\text{Cu}}^{+}+{\text{O}}_{2}+{\text{OH}}^{•}+{\text{H}}_{2}\text{O}\\ {\text{Cu}}^{+}+{\text{H}}_{2}{\text{O}}_{2}⟶{\text{Cu}}^{2+}+{\text{OH}}^{•}+{\text{OH}}^{-}\\ \text{Over}\;\text{all:}\;3{\text{H}}_{2}{\text{O}}_{2}⟶{\text{O}}_{2}+2{\text{OH}}^{•}+{2\text{H}}_{2}\text{O} \end{array}$$

Retinol (analyte) can go through electrons transfer reactions being n-electron donor as reported [30] and cannot quench singlet oxygen and have only very weak capacity to scavenge free radicals [1]. In the proposed study, the analyte which acts as an electron donor, transfer electrons to the lowest triplet excited state of RB, generating anion radicals of RB. These anion radicals of RB act as reducing radicals and react rapidly with dissolved oxygen to form superoxide anion and via its dismutation H_2_O_2_ is formed. Copper which acts as a catalyst generates highly reactive ^•^OH radicals through the classical fenton reaction with H_2_O_2_. These highly reactive radicals oxidize luminol and generate chemiluminescence. Based on the above discussion the possible reaction scheme for the proposed FI-CL method can be summarized as

(7) $$\begin{array}{c} \text{RB}+h\nu ⟶{\text{RB}}^{*}\left(\text{T}\right) \end{array}$$

(8) $$\begin{array}{c} {\text{RB}}^{*}\left(\text{T}\right)+{\text{e}}^{-}\;\left(\text{analyte}\right)⟶{\text{RB}}^{•-} \end{array}$$

(9) $$\begin{array}{c} {\text{RB}}^{•-}+{\text{O}}_{2}⟶{{\text{O}}_{2}}^{•-}+\text{RB} \end{array}$$

(10) $$\begin{array}{c} {{2\text{O}}_{2}}^{•-}+2{\text{H}}^{+}⟶{\text{H}}_{2}{\text{O}}_{2}+{\text{O}}_{2} \end{array}$$

(11) $$\begin{array}{c} {\text{Cu}}^{2+}+{\text{H}}_{2}{\text{O}}_{2}⟶{\text{Cu}}^{2+}+{\text{O}}_{2}+{2\text{OH}}^{•}+{2\text{H}}_{2}\text{O} \end{array}$$

(12) $$\begin{array}{c} {\text{Luminol}+{\text{O}}_{2}}^{•-}/{2\text{OH}}^{•}⟶\text{Aminophthalate}+h\nu \;\left(425\;\text{nm}\right) \end{array}$$

### 3.3. Figures of Merit

The method is successfully applied to the determination of vitamins A and C and RB in pharmaceutical formulations and results are given in Table 2. The interference of foreign substances/common excipients found in pharmaceutical formulations was examined by analyzing a standard solution of both vitamins (0.3 mg/L) into which increasing amounts (25-, 50-, and 100-fold) of interfering analyte were added. The tolerance of each foreign species was taken as the largest concentration giving less than ±5% error of the added concentration of vitamin. No interference could be found with 100-fold for Na^+^, K^+^, Ca^2+^, Mg^2+^, Zn^2+^, NH_4_^+^, SO_4_^2−^, PO_4_^3−^, NO_3_^−^, HCO_3_^−^, Cl^–^, glycerol, cholesterol, stearic acid, methyl cellulose, glucose, sucrose, starch, gum acacia, magnesium stearate, vitamins D, K_1_, and K_2_, mannitol, oleic acid, polyvinyl alcohol, and albumin.

## 4. Conclusions

A simple FI-CL method has been established for the determination of vitamins A and C using RB as a photosensitizer for the first time. The method was successfully applied to the determination of retinol, vitamin C, and RB in pharmaceutical formulations and the results obtained gave good agreement between the results obtained and values labeled. We also proposed the scheme for RB sensitization effect on chemiluminescence.

## Figures and Tables

**Table 1:** Selection of variables.

Variables	Range studied	Optimum
Pyrophosphate buffer pH (0.01 M)	8.5–11.0	10.25
Rose Bengal (M)	1 × 10^−5^–2.5 × 10^−4^	1 × 10^−4^
Copper(II) (M)	1 × 10^−4^–1 × 10^−3^	5 × 10^−4^
Luminol (NaOH 0.12 M)	1 × 10^−6^–1 × 10^−5^ M	5 × 10^−5^ M
Total flow rate (mL min^−1^)	2–10	6.0
Sample volume (μL)	60–300	60
Photoreactor coil length (m)	0.5–3.0	2.0

**Table 2:** Analytical performance and analyte determination in pharmaceuticals by the proposed method (*n* = 5).

Figures of merit	Vitamin A	Vitamin C	Rose Bengal
Calibration equation [*I*= intensity, *c* = concentration (μg/mL)]	*I* = 67.88*c* + 2.62	*I* = 70.15*c* + 2.91	*I* = 47.98*c* + 1.71
Linear range (μg/mL)	0.05–15	0.01–20	0.1–50
Limit of detection (μg/mL, *S*/*N* = 3)	0.008	0.005	0.05
Sample throughput	70	70	30

Pharmaceutical formulations	Proposed method results/ (labeled values) (μg)	Proposed method results/ (labeled values) (mg)	Proposed method results/ (labeled values) (%)

Capsules	210 ± 3/(200)	—	—
Chewable dragee	9975 ± 5/(9900)	97 ± 6/(100)	—
Eye drops (0.6 mL)	—	—	1 ± 0.1/(1)
Injections	3378 ± 4/(3300)	—	—
Tablets	—	509 ± 5 mg/(500)	—
